# Whole genome resequencing analysis of tobacco K326 and its cold sensitive mutant M18

**DOI:** 10.3389/fpls.2026.1829823

**Published:** 2026-05-01

**Authors:** Hui Yin, Xiuping Li, Yue Wang, Jianlin Wang, Zhengyu Deng, Zhimin Chen, Haocun Tang, Luping Zhu, Risheng Hu, Zhengrong Hu

**Affiliations:** 1 Hunan Tobacco Research Institute, Changsha, China; 2 College of Agriculture, Hunan Agricultural University, Changsha, China; 3 College of Life Sciences, Hunan Normal University, Changsha, China; 4 Yongzhou Tobacco Company, Yongzhou, China

**Keywords:** early flowering, KEGG enrichment analysis, low temperature stress, tobacco, whole genome resequencing

## Abstract

**Introduction:**

Low temperatures cause flower prematurely of tobacco sensitive variety, severely impacting its yield and quality.

**Methods:**

To uncover the molecular mechanisms underlying cold-induced early flowering in tobacco, the whole-genome resequencing was performed on the wild-type K326 and its cold-sensitive mutant M18.

**Results:**

Compared with the reference genome,1,155,183 and 1,724,339 single nucleotide polymorphisms (SNPs), 286,674 and 360,131 small fragment insertion/deletion sites (InDels) were detected in wild-type K326 and its mutant M18. There are 5,735 and 14,892 nonsynonymous mutations in the coding region, causing a total of 5,588 gene mutations. The GO classification annotation of genes containing SNP/Indel sites shows that a relatively large number of genes are annotated to categories such as plant reproduction, metal ion transport, plant systemic cell formation, nucleotide binding, kinase activity, and tRNA binding. KEGG analysis indicated that the genes were mainly involved in the pathways of plant metabolic pathway, oxidative phosphorylation and plant photosynthesis. Through comparative analysis of SNP and InDel differences between the two materials, 75 candidate genes associated with flowering and stress resistance were identified, including transcription factors (MYB, WRKY) and key kinases (14-3-3, MAPK, CDPK).

**Discussion:**

These selected genes will not only facilitate understanding of genetic basis of cold stress response, but also accelerate genetic improvement through marker-assisted selection in tobacco.

## 1 Introduction

The transition from vegetative to reproductive growth is a critical phase in the plant life cycle. It is regulated not only by endogenous development signals of plant, but also external environmental factors. Six pathways are well known of flower regulation, including photoperiod, vernalization, gibberellin, autonomy, temperature sensitivity and age pathway (Wellmer and Riechmann, 2010). These pathways perceive and transmit flowering signals, and initiate flowering by regulating two opposite MADS-box genes. One is the strong flowering inhibitor FLOWERING LOCUS C (*FLC*) and SHORT VEGETA-TIVE PHASE (*SVP*); the other is the flowering integration factor (*FT* and *SOC1*) and flower primordium characteristic gene (*AP1, LFY, CAL, FUL, AGL24*), which are MADS-box genes except *FT* and *LFY*. FLC could directly inhibit the transcriptional expression of *FT* and *SOC1* to inhibit flowering (Sharma et al., 2017, Sharma et al., 2020). Florigen (FT) is a signal that transmits light stimulation-induced flowering in the photoperiod pathway. *FT* gene encodes phosphatidyl ethanolamine-binding protein (PEBP) which is transported to the top of the stem to activate the expression of AP1 (Hong et al., 2020). In the apical meristem, FT protein interacts with FD (flowering locus D) and 14-3–3 protein forms a complex to induce the expression of the downstream flowering genes, thereby promoting flowering (Taoka et al., 2011). There are 12 PEBP family members in tobacco, including 5 FT-like and 7 TFL1-like subfamily members (Karlgren et al., 2011). Among *FT-like* subfamily members in tobacco, the *NtFT1, NtFT2* and *NtFT3* are floral inhibitors, whereas *NtFT4* and *NtFT5* are floral inducers. It was found that the expression level of *NtFT4* was much lower than that of *NtFT1* and *NtFT2* at the seedling stage, but the expression level of *NtFT4* was significantly higher than that of *NtFT1* and *NtFT2* at the flowering stage (Harig et al., 2012). The members of TFL1-like subfamily in tobacco include *NtCET1, NtCET2, Nt-CET4* and *NtTFL1–4* genes (Wang et al., 2018).

Cold stress is a major abiotic stress, which severely limited plant growth and development. The well-established cold response pathway of plant is the ICE–CBF–COR signaling cascade (Wang et al., 2019). *ICE*, a pioneer transcription factor in the ICE-CBF-COR signaling cascade, acts upstream of cold-regulated genes. Upon exposure to cold stress, Ca²^+^ ions activate CRLKs (Calcium/Calmodulin-Regulated Receptor-Like Kinases) in the calcium signaling pathway, which subsequently trigger the MAPK cascade through MPK3 and MPK6. *ICE1*, a member of the bHLH family of transcription factors, can regulate the expression of cold-induced genes. ICE proteins induce the expression of *CBF* by binding to the *MYC* cis-acting elements in the promoter of CBF3/DREB1A (Zhou et al., 2021). *CAMTA3* family transcription factors, the key components of the Ca²^+^ signaling pathway, are regulated by MAPK genes, and directly suppress the expression of *ICE* or activate the expression of *CBF*, thereby to enhance cold tolerance through regulating *COR* genes (Meng et al., 2021; Hwarari et al., 2022; Tang et al., 2020).

Whole genome resequencing refers to sequencing the genomes of different individuals of a specie with a known genome sequence, and then conducting the sequence differences analysis on individuals or populations. It is used to assist in the discovery of single nucleotide polymorphism (SNP), insertion and deletion (InDel), structural variation (SV) and copy number variation (CNV). Through bioinformatics methods, differences and structural variations in gene sequences can be explored, structural differences between the genomes of different individuals can be analyzed, and annotations can be completed (Xu et al., 2023).

Tobacco, belonging to *Solanaceae*, is a thermophilic crop. Continuous low temperature stress can cause early flowering in tobacco, which correspondingly leads to reduced plant height, fewer leaves, narrower leaves, and seriously affects its yield and quality (Hu et al., 2022). Whole-genome resequencing of a common cultivar K326 and its cold-sensitive mutant M18 was conducted to comprehensively reveal genomic variations between the two varieties, and then to further explore the key candidate genes. These genes could be the fundamental genetic resource for enhancement of tobacco cold tolerance, and provide insight into molecular mechanisms of cold-induced early flowering of tobacco.

## 2 Materials and methods

### 2.1 Plant materials and treatments

The test materials were common cultivar K326 (WT) and its cold-sensitive mutant M18. The cold-sensitive mutant M18 was derived from K326 by EMS mutagenesis to construct a mutant library. Approximately 1,000 seeds were selected from the bank and germinated in the soil. When the seedlings grew to the six-leaf and one-heart stage, they were treated at a low temperature of 10 °C for 14 days. After transplanting, flowering time was investigated under simulated field conditions, and one stable line showing approximately 10 days earlier flowering than WT was obtained. After three consecutive generations of self-pollination, the homozygous and genetically stable mutant line M18 was finally obtained. A total of four groups were set up as follows:CK-K326 (control at normal temperature), CK-M18 (control at normal temperature), LT-K326 (low-temperature treatment for 14 days), and LT-M18 (low-temperature treatment for 14 days). Then the seedlings were divided into two groups, both of which were transferred to normal growth conditions for continued cultivation. One group was used for the investigation of indicators such as flowering period and days required for bud formation; and the other group of seedlings was used for gene expression sampling. The samples were respectively collected at 7 days and 14 days after low-temperature (10 °C) treatment, and 10 days and 20 days after recovery at room temperature (25 °C) (A: treatment for 7 days; B: treatment for 14 days; C: recovery 10 days; D: recovery 20 days).

### 2.2 Whole genome resequencing

#### 2.2.1 Extraction of total DNA and library construction

Large-scale extraction was performed according to CTAB method (Cetyl trimethyl ammonium bromide, CTAB). The quality and integrity of the DNA were assessed via agarose gel electrophoresis, while the DNA concentration was determined and accurately quantified using Nanodrop and Qubit 3.0. High-quality DNA samples were randomly fragmented into 350 bp segments using Covaris, followed by library construction. The DNA fragments underwent terminal repair, the addition of polyA tails, incorporation of sequencing adapters, purification, PCR amplification, and other necessary steps, culminating in the completion of the library construction. Upon completion, Qubit 3.0, Agilent 2100, and qPCR were employed to detect and accurately quantify the library (> 2 nM) to ensure its quality. Double-ended sequencing was conducted using the PE150 sequencing strategy on the Illumina platform.

#### 2.2.2 Sequence alignment, SNP, InDel, SV and CNV detection and annotation

The valid data was aligned to the reference genome using BWA software, and duplicate alignment results were removed with SAMTOOLS (Li and Durbin, 2009) (Li et al., 2009). Subsequently, SNPs and InDels were analyzed and annotated using ANNOVAR software, while CNVs were analyzed and annotated with CNVnator (parameter: -call 100) software. Structural variants (SVs) were analyzed and annotated by using BreakDancer software. SNP and InDel filtering criteria: variants were filtered with the following thresholds: QUAL≥20, DP≥4, QD≥2.0, FS≤60.0, MQ≥40.0, and SOR ≤3.0.

#### 2.2.3 Differential gene functional annotation

Functional enrichment analysis was performed on genes with non-synonymous mutations across samples using the R package ClusterProfiler. This analysis included enrichment assessments for both GO annotations and KEGG pathways, with gene annotations determined following false discovery rate (FDR) multiple testing correction. Enrichment significance thresholds: P-value < 0.05 and FDR < 0.10 were used as the cutoffs to identify significantly enriched terms and pathways.

### 2.3 Investigating agronomic traits

(20 replicates per group) The plants (20 biological replicates per group) were transferred to normal conditions for continued cultivation. During this period, the plant phenotypes were continuously observed, and photographs were taken when the seedlings were 90 days old. Meaning while, the number of days were recorded from sowing to first flower opening for each group of seedlings.

### 2.4 Gene expression analysis

#### 2.4.1 Extraction of total RNA and cDNA synthesis

Total RNA in each sample was extracted using an FastPure^®^ Universal Plant Total RNA Isolation Kit (RC411-01; Nanjing Vazyme Biotech Co., Ltd. Nanjing, China), then the corresponding cDNA was synthesized using the HiScript^®^ III; 1st Strand cDNA Synthesis Kit (+gDNA wiper) (R312-01/02; Nanjing Vazyme Biotech Co., Ltd. Nanjing, China).

#### 2.4.2 qRT-PCR analysis

The qRT-PCR analysis was carried out using the QuantStudioTM 3 Real-Time PCR Instrument (Applied Biosystems, USA) with the following parameters:95 °C for 30 s, followed by 40 cycles of 95 °C for 5 s and 58 °C for 30 s, then 95 °C 15 s, 60 °C for 1 min, and 95 °C for 15 s. The reaction system consists of 5 uL of PowerUpTM SYBRTMGreen Master Mix, 0.2 uL of each upstream and downstream primer, 1 µL of cDNA, and 3.6 uL of ddH_2_O. *NtActin* was used as the internal control. Primer information for qPCR is shown in **Table 1**.

**Table 1:** qRT-PCR primer sequence information.

Primer name	Primer sequences (3’→5’)
*NtActin*-q-F	TGCTGATCGTATGAGCAAGG
*NtActin*-q-R	ATCCTCCGATCCAGACACTG
*NtFT4*-q-F	GATATCCCAGCAACTACAGATACAAG
*NtFT4*-q-R	GAAACGGGCAAACCAAGATTGTAAAC
*NtFT5*-q-F	TGCCAAGAGAACGTGAACCT
*NtFT5*-q-R	GGCCTAAGCTCACACCCATT

### 2.5 Statistical analysis

In this present study, each experiment was performed with at least three biological replicates. Statistical significance were compared or by one-way ANOVA followed by Tukey’s test.

## 3 Results

### 3.1 Analysis of flowering time and flowering regulatory gene expression following low-temperature stress

**Figure 1:**
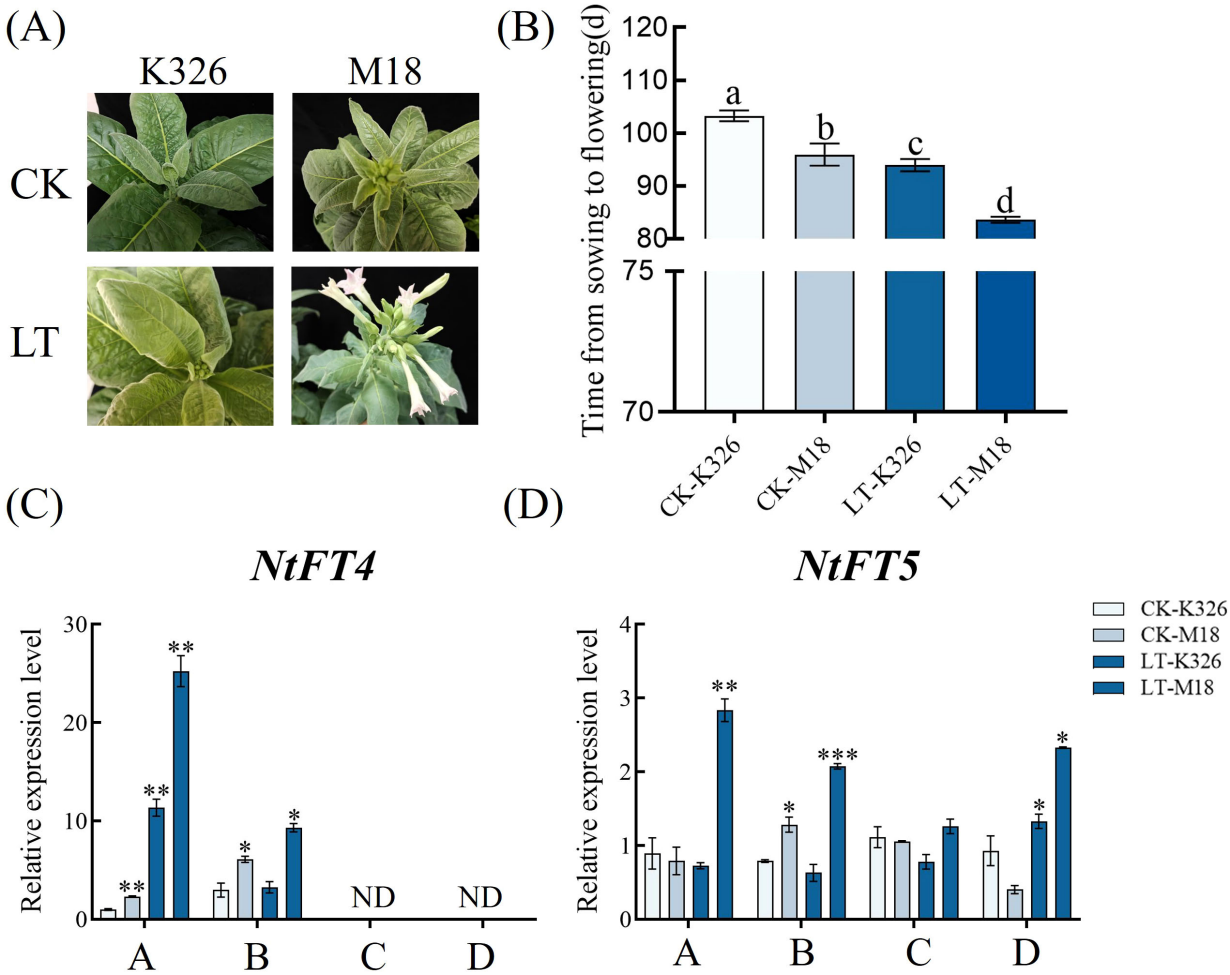
Analysis of flowering period and gene expression in flowering regulation. **(A)** Flowering phenotype at 90 days of seedling age. **(B)** Number of days from sowing to the opening of the central flower. **(C)** NtF4 and **(D)** NtFT5 gene expression analysis. Each group has at least 3 biological replicates. Different letters indicate significant differences with P < 0.05; * indicates differences between different groups under the same condition, with P < 0.05 (*), P < 0.01 (**), P < 0.001 (***) P<0.05(*), P<0.01(**), P<0.001(***). ND, not detected.

As shown in **Figure 1A**, under both control and low-temperature conditions, M18 shows flower bud formation or flowering significantly earlier than K326, and low-temperature stress exacerbates the difference between the two varieties. The results of the flowering period survey show that M18 blooms earlier than K326 and is more sensitive to low temperatures (**Figure 1B**). Furthermore, we analyzed the expression of several key flowering regulatory genes. *NtFT4* can be detected at time points A and B, but not at time points C and D. Under the same conditions, the expression level of *NtFT4 i*n M18 is significantly higher than that in K326. Low-temperature stress leads to a significant upregulation of *NtFT4* expression in M18, with the peak reached at time point A. Differently, *NtFT5* could be detected at all four time points. Low-temperature stress significantly upregulated the expression level of this gene in M18, while there was little change in K326. Moreover, at the same time point, the expression level of *NtFT5* in LT-M18 was significantly higher than that in the other three groups.

### 3.2 Analysis of the results compared with the reference genome

The raw sequencing data for K326 and its mutant M18 yielded 135,695,988,300 and 135,687,904,200 reads, respectively. After filtering, 134,594,705,400 and 134,744,239,500 high-quality reads were retained, with valid read ratios of 99.13% and 99.31%, respectively. The GC content, ranging from 39.09-39.48% was moderate, and the sequencing results of Q20>96% and Q30>90% were considered high-quality (**Supplementary Table 1**). These results indicate that all samples possessed sufficient sequencing depth, met quality standards, and displayed normal GC distribution. The high-quality clean reads from K326 and M18 were independently aligned to the reference genome, achieving mapping rates exceeding 99% for both genotypes. The average coverage depth across the reference was 24.44× for K326 and 25.71× for M18. Furthermore, the proportion of reference sites covered by at least one base was above 98.14%, while sites covered by at least four bases reached over 92.21% (**Supplementary Table 2**). These data demonstrated successful alignment, high coverage uniformity, and sufficient sequencing quality of the resequencing, supporting the reliability of subsequent genomic and comparative analyses.

### 3.3 SNP genotype detection and annotation

#### 3.3.1 Analysis of SNP distribution and mutation patterns

**Figure 2:**
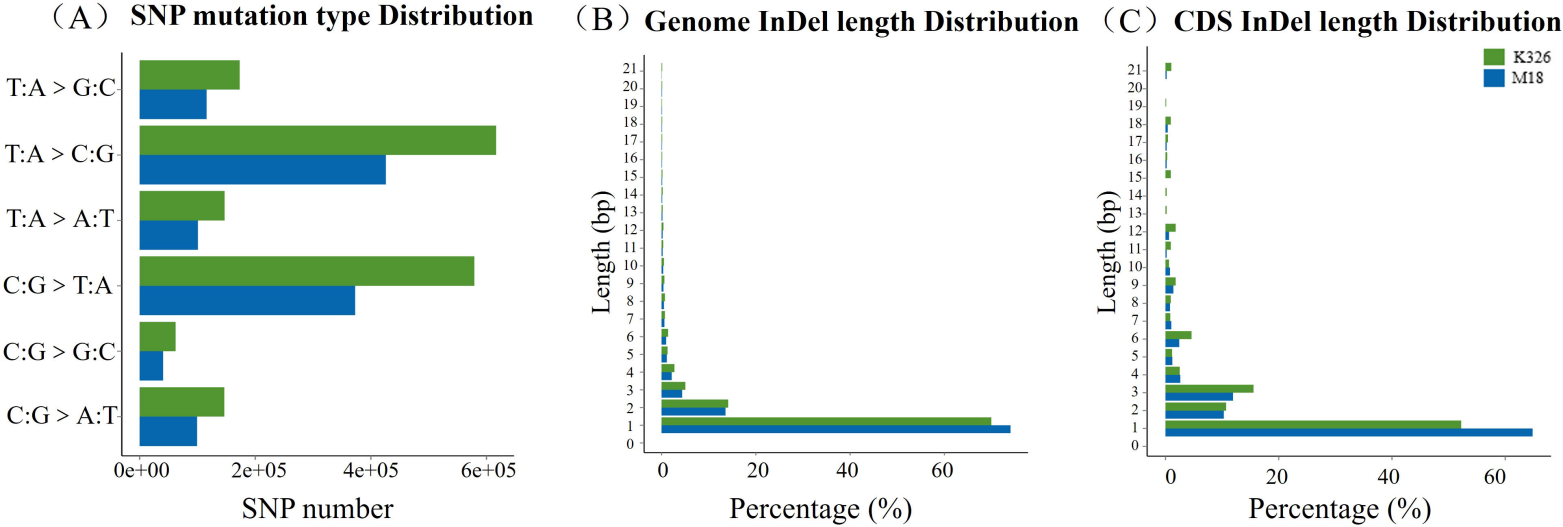
SNP mutation types **(A)**; genome-wide **(B)** and CDS **(C)** InDel length distributions.

The distribution of SNPs was analyzed across genomic regions between K326 and M18 (**Supplementary Table 3**) and their mutational patterns (**Figure 2A**, **Supplementary Table 4**). Comparison with the reference genome, a total of 1,155,183 SNP loci was detected in K326, with 798,758 transitions, 356,425 transversions, and heterozygous ratio of 0.257. A total of 1,120,695 (97.01%) SNP sites were located within gene regions, with 5,735 (1.5%) SNP sites in exon regions affecting gene function. Furthermore, 1,724,339 SNPs were identified in M18, with 1,195,771 transitions, 528,568 transversions, and heterozygous ratio of 0.272. The majority of these variants (1,624,272;94.20%) were located within genic regions. Among these, 14,829 SNPs (accounting for 0.86%) were detected in exons, which were capable of altering gene function. Notably, there were more exonic SNPs in M18 more than those of K326.

#### 3.3.2 Functional annotation and statistics of SNPs in coding regions

Mutations in exons were classified and annotated according to the alteration in amino acids (**Table 2**). Typically, nonsynonymous mutations lead to changes in the corresponding amino acids. In addition, the gain and loss of stop codons could result to obvious changes in translation and loss-function of proteins. The three variant types differ by 5,449 loci, which may be contributed to the different cold responses between the K326 and its M18 mutation.

**Table 2:** Coding region SNP variant types.

Category	K326	M18	Difference
Exonic	5735	14892	9157
Exonic Stop gain	83(1.45%)	194(1.30%)	111
Exonic Stop loss	37(0.65%)	52(0.35%)	15
Exonic Synonymous	2219(38.69%)	5927(39.80%)	3708
Exonic Non-synonyous	3396(59.22%)	8719(58.55%)	5323
Exonic unknowns	0	0	0

### 3.4 InDel genotype detection and annotation

#### 3.4.1 Analysis of InDel distribution and mutation patterns

The genomic distribution of annotated InDels was analyzed of K326 and M18 (**Supplementary Table 3**). Comparison with the reference genome, a total of 286,674 InDel loci were found in K326. The vast majority of these InDels (261,643, 91.26%)were located within gene regions. Notably, 469 InDels(0.61%)were identified in exonic regions. A total of 360,131 InDel loci were identified in M18. Among these, 321,791 InDels(89.35%)were located within gene regions, and 925(0.26%)were identified in exonic regions. The results indicate that significantly fewer InDel variants occur in coding regions compared to non-coding regions.

#### 3.4.2 Functional annotation and statistics of InDels in coding regions

K326 and M18 were annotated and classified according to whether the mutations in exons caused amino acid changes (**Table 3**). Typically, insertions or deletions in exons that involve a number of bases not divisible by three (frameshift mutations) may alter the protein reading frame, thereby affecting gene function. Similarly, both the gain and loss of stop codons could lead to premature termination of translation. A difference of 278 sites was observed in these four variant types between the K326 and M18, suggesting potential functional divergence in their respective genes.

**Table 3:** Coding region indel variant types.

Category	K326	M18	Difference
Exonic	469	925	456
Exonic Stop gain	4(0.85%)	13(1.41%)	9
Exonic Stop loss	3(0.64%)	7(0.76%)	4
Exonic Frameshift deletion	128(27.29%)	279(30.16%)	151
Exonic Frameshift insertion	250(53.3%)	364(39.35%)	114
Exonic Non-frameshift deletion	49(10.45%)	128(13.84%)	79
Exonic Non-frameshift insertion	35(7.26%)	134(14.49%)	99

#### 3.4.3 InDel mutation length distribution statistics

The distribution of InDel with different mutation lengths in the coding region and the whole genome was similar of K326 and M18 plants (**Figures 2B, C**). The proportion of 1 bp InDel mutation in the coding region was the highest of both K326 and M18, and the value of K326 was high than that of M18. Besides, the proportion of 2 bp InDel mutation in the coding region was no obvious different between the two plant materials.

### 3.5 SV/CNV genotype detection and annotation

SV variation structure includes four categories, namely INS (Insertion), DEL (Deletion), INV (Inversion), ITX (Intra-chromosomal translocation) and CTX (Inter-chromosomal translocation). A total of 28,407 SV variation sites were found in K326, of which 12356 (88.48%) were located in the gene region, and 453 (3.11%) SV variation sites were located in the exon region affecting gene function (**Supplementary Table 3**; **Supplementary Figures 1A, B**). A total of 30726 SV variation sites were found in M18, of which 14,132 (87.27%) variation sites were located in the gene region, 600 (3.71%) SV variation sites were located in the exon, and the distribution of the remaining variation sites was basically the same as that of K326. Among them, the main variation types are DEL and ITX.

CNV variation structure includes two categories:Gain number and Loss number. CNV results showed that (**Supplementary Table 3**; **Supplementary Figures 1C, D**), compared with the reference genome,106,307 CNV variation sites were found in K326, 106,878 SV variation sites were found in M18, the distribution of the two variation types in K326 and M18 was basically the same.

### 3.6 Identification and functional annotation of variant genes

**Figure 3:**
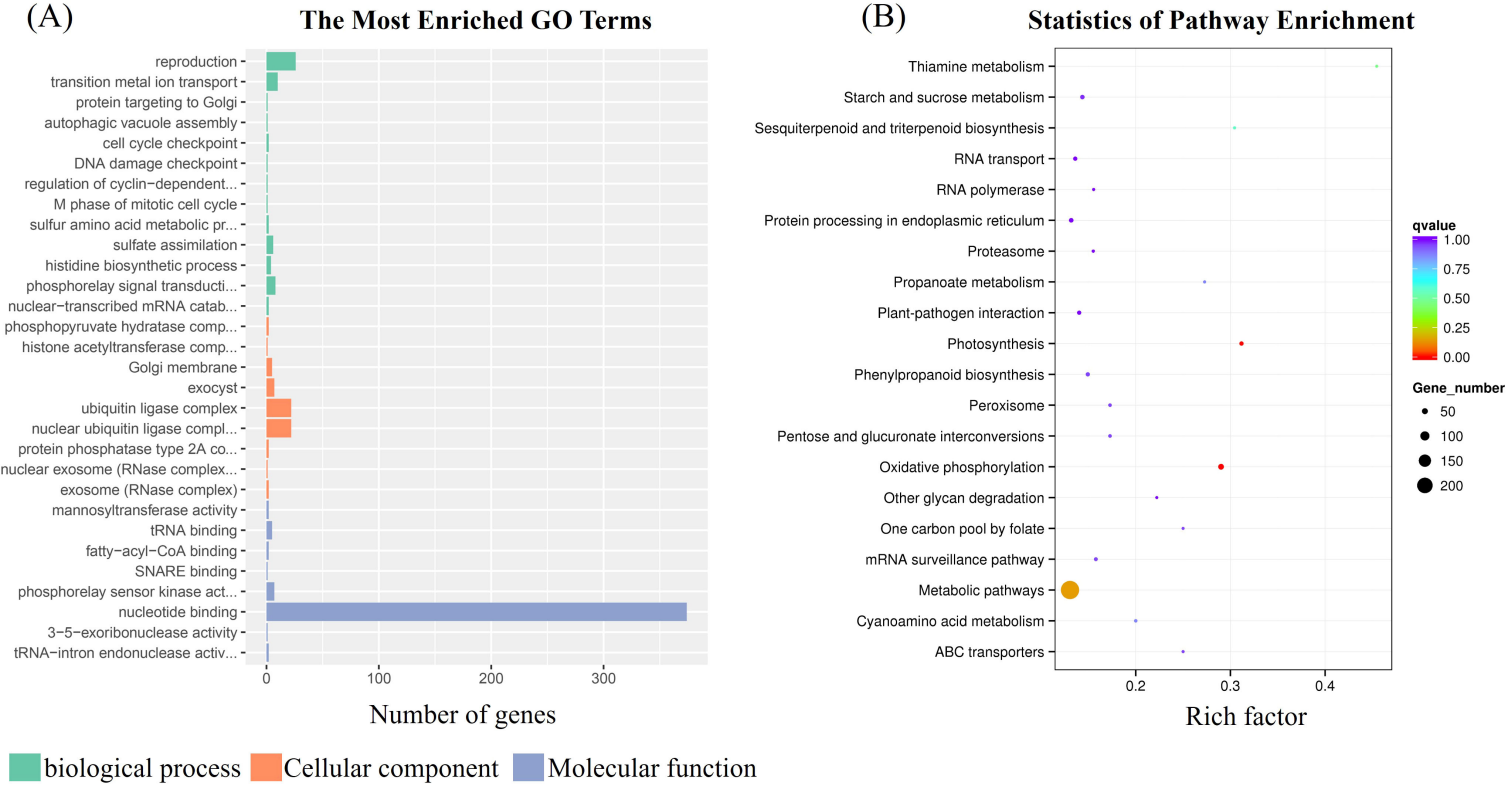
GO enrichment analysis **(A)** and KEGG enrichment analysis **(B)** of the the variant genes.

GO annotation and enrichment analysis were performed of mutant genes at SNP and InDel sites in the CDS region of K326 and its mutant M18 (**Figure 3A**). In the biological process category, the genes with mutated loci are mainly enriched in plant reproduction, metal ion transport, etc. In the cell component category, the mutant genes are mainly related to the formation of plant whole-body cells; in the molecular function category, the mutant genes are mainly related to nucleotide binding, kinase activity and tRNA binding. In addition, KEGG enrichment analysis displayed that these genetic variations were mainly involved in the pathways of plant metabolic pathways, oxidative phosphorylation and plant photosynthesis (**Figure 3B**).

In order to further explore the mutation genes related to the flowering delay and low temperature tolerance of mutant M18 at the DNA level, 75 genes were found to directly or indirectly affect the flowering and stress resistance process of plants by combining KEGG, GO with SNP and InDel, respectively. Among them, 63 variable genes belong to SNP locus variation (**Table 4**), and 12 variable genes belong to InDel locus variation (**Table 5**), including *WD40, MYB, bHLH*, *WRKY* and *BZR1* transcription factors;14-3–3 protein and MAPK, CDPK and other protein kinases.

**Table 4:** Candidate genes for SNP site variations.

Gene ID	Annotations	K326 genotype	Ref/Alt depth	Quality	M18 genotype	Ref/Alt depth	Quality	M18 type
Nitab4.5_0000604g0030.1	GH3 auxin-responsive promoter	AA	21|0	285	GG	0|16	225	stoploss
Nitab4.5_0000617g0250.1	LRR, Leucine-rich repeat	TT	26|0	285	GG	0|19	225	
Nitab4.5_0000005g0190.1	DNA-binding WRKY	GG	22|0	285	AA	0|18	192	stopgain
Nitab4.5_0005731g0030.1	Zinc finger	GG	23|0	285	AA	0|19	225	
Nitab4.5_0001544g0170.1	Zinc finger	CC	21|0	285	AA	0|22	225	
Nitab4.5_0000046g0010.1	Zinc finger	AA	14|0	187	TT	0|9	107	
Nitab4.5_0001140g0090.1	Serine/threonine-protein kinase	AA	18|0	285	CC	0|22	225	
Nitab4.5_0001545g0010.1	LRR, Leucine-rich repeat	CC	19|0	285	TT	0|23	225	
Nitab4.5_0002334g0090.1	14-3–3 protein	CC	20|0	285	AA	0|5	119	
Nitab4.5_0005819g0010.1	Myb domain	GG	17|0	285	AA	0|25	225	
Nitab4.5_0006378g0030.1	WD40 repeat	CC	28|0	285	TT	0|23	225	
Nitab4.5_0000578g0020.1	Zinc finger\WD40 repeat	TT	20|0	285	AA	0|24	225	nonsynonymous SNV
Nitab4.5_0000837g0070.1	Zinc finger\WD40 repeat	GG	24|0	285	AA	0|22	225	
Nitab4.5_0000578g0020.1	Zinc finger\WD40 repeat	TT	20|0	285	AA	0|24	225	
Nitab4.5_0001765g0050.1	MYC/MYB \bHLH	CC	19|0	285	TT	0|30	225	
Nitab4.5_0002539g0040.1	MYC/MYB \bHLH	CC	17|0	285	TT	0|17	225	
Nitab4.5_0000036g0090.1	Myb domain	AA	26|0	285	CC	0|23	225	
Nitab4.5_0000073g0400.1	Sucrose synthase	TT	28|0	285	GG	0|20	225	
Nitab4.5_0007169g0010.1	Sucrose synthase	AA	22|0	285	TT	0|20	225	
Nitab4.5_0000628g0120.1	BZR1, transcriptional repressor	AA	24|0	285	GG	0|15	225	
Nitab4.5_0000790g0070.1	BZR1, transcriptional repressor	AA	19|0	285	TT	0|23	225	
Nitab4.5_0004702g0020.1	DNA-binding WRKY	GG	14|0	285	AA	0|27	225	
Nitab4.5_0000274g0220.1	ZF-HD homeobox protein	TT	21|0	285	AA	0|21	225	
Nitab4.5_0000090g0240.1	Zinc finger	CC	23|0	285	TT	0|21	225	
Nitab4.5_0001541g0040.1	Zinc finger	GG	22|0	285	AA	0|27	225	
Nitab4.5_0002616g0040.1	Zinc finger	GG	27|0	285	TT	0|23	225	
Nitab4.5_0004190g0020.1	Zinc finger	CC	26|0	285	GG	0|27	225	
Nitab4.5_0006935g0020.1	Zinc finger	CC	24|0	285	TT	0|21	225	
Nitab4.5_0000628g0080.1	bZIP,G-box binding	GG	26|0	285	CC	0|31	225	
Nitab4.5_0000235g0140.1	Zinc finger	GG	21|0	285	TT	0|11	134	
Nitab4.5_0000467g0100.1	Zinc finger	TT	27|0	285	CC	0|20	225	
Nitab4.5_0001002g0070.1	bZIP,G-box binding	CC	24|0	285	AA	0|26	225	
Nitab4.5_0000265g0070.1	UDP-glucose/GDP-mannose	TT	29|0	285	AA	0|24	225	
Nitab4.5_0000212g0180.1	UDP-glucose/GDP-mannose	CC	22|0	285	GG	0|21	219	
Nitab4.5_0000101g0350.1	Protein kinase domain	GG	42|0	285	AA	0|23	225	
Nitab4.5_0000272g0150.1	Protein kinase domain	GG	23|0	285	TT	0|23	225	
Nitab4.5_0013081g0010.1	Protein kinase domain	TT	21|0	285	CC	0|21	225	
Nitab4.5_0000072g0550.1	Serine/threonine-protein kinase	AA	25|0	285	TT	0|24	225	
Nitab4.5_0000136g0460.1	catalytic domain	CC	29|0	285	AA	0|30	225	
Nitab4.5_0000268g0080.1	Serine/threonine-protein kinase	GG	23|0	285	AA	0|27	225	
Nitab4.5_0000297g0290.1	Serine/threonine-protein kinase	TT	23|0	285	CC	0|26	225	
Nitab4.5_0000319g0240.1	Serine/threonine-protein kinase	CC	29|0	285	AA	0|20	225	
Nitab4.5_0000467g0070.1	Serine/threonine-protein kinase	GG	24|0	285	AA	0|26	225	
Nitab4.5_0001180g0290.1	Serine/threonine-protein kinase	AA	24|0	285	GG	0|20	225	
Nitab4.5_0002113g0210.1	Serine/threonine-protein kinase	CC	22|0	285	TT	0|27	225	
Nitab4.5_0002579g0050.1	Serine/threonine-protein kinase	GG	21|0	285	CC	0|24	225	
Nitab4.5_0002766g0080.1	Serine/threonine-protein kinase	GG	20|0	285	AA	0|26	225	
Nitab4.5_0003540g0130.1	Serine/threonine-protein kinase	TT	21|0	285	CC	0|25	225	
Nitab4.5_0005615g0040.1	Serine/threonine-protein kinase	TT	20|0	285	AA	0|22	225	
Nitab4.5_0006786g0020.1	Serine/threonine-protein kinase	TT	21|0	285	CC	0|23	225	
Nitab4.5_0024282g0010.1	Serine/threonine-protein kinase	GG	21|0	285	AA	0|22	225	
Nitab4.5_0001663g0190.1	Serine/threonine-protein kinase	AA	23|0	285	TT	0|25	225	
Nitab4.5_0001060g0060.1	CDPK	CC	24|0	285	AA	0|32	225	
Nitab4.5_0000022g0300.1	MAPK	AA	22|0	285	TT	0|31	225	
Nitab4.5_0000076g0230.1	LRR, Leucine-rich repeat	TT	27|0	285	CC	0|25	225	
Nitab4.5_0000586g0040.1	LRR, Leucine-rich repeat	GG	25|0	285	CC	0|25	225	
Nitab4.5_0001056g0010.1	LRR, Leucine-rich repeat	GG	36|0	285	AA	0|21	225	
Nitab4.5_0002405g0040.1	LRR, Leucine-rich repeat	AA	22|0	285	CC	0|27	225	
Nitab4.5_0004398g0010.1	LRR, Leucine-rich repeat	AA	23|0	285	CC	0|21	225	
Nitab4.5_0000070g0010.1	Disease resistance protein	AA	25|0	285	GG	0|23	225	
Nitab4.5_0010433g0010.1	Disease resistance protein	AA	20|0	285	CC	0|20	225	
Nitab4.5_0021935g0010.1	Disease resistance protein	GG	45|0	285	AA	0|23	225	
Nitab4.5_0019677g0010.1	Late blight resistance protein R	GG	32|0	285	AA	0|30	225	

The colored values represent the sequencing depths of the reference (Ref) and alternative (Alt) alleles at each SNP locus within the flowering- and stress resistance-related candidate genes, as detected in the corresponding samples.

**Table 5:** Candidate genes for InDel site variations.

Gene ID	Annotations	K326 genotype	Ref/Alt depth	Quality	M18 genotype	Ref/Alt depth	Quality	M18 type
Nitab4.5_0000363g0330.1	Zinc finger	TG TG	15|0	285	TGTCCAAGAACCCTTCAGAA GTGTCCAAGACCCTTCAGAAG	0|19	225	stopgain
Nitab4.5_0000046g0010.1	Zinc finger	TCC TCC	21|0	285	TC TC	0|27	225	frameshift deletion
Nitab4.5_0000171g0280.1	Zinc finger	A A	15|0	285	ACACCCCAACC CACACCCCAACCC	0|12	225	
Nitab4.5_0001004g0150.1	Zinc finger	CAA CAA	25|0	285	CA CA	0|31	225	
Nitab4.5_0001274g0020.1	Serine-threonine/tyrosine-protein kinase catalytic domain	GAA GAA	23|0	285	GA GA	0|24	225	
Nitab4.5_0009785g0050.1	Serine-threonine/tyrosine-protein kinase catalytic domain	ACCCCCCCC ACCCCCCCC	19|0	285	ACCCCCCCCC ACCCCCCCCC	0|13	64	
Nitab4.5_0001180g0230.1	Serine/threonine-/dual specificity protein kinase	GTTTTTT GTTTTTT	13|0	285	GTTTTTTT GTTTTTTT	0|24	130	
Nitab4.5_0004268g0030.1	Serine/threonine-/dual specificity protein kinase	CTTTTTT CTTTTTT	24|0	285	CTTTT CTTTT	0|22	225	
Nitab4.5_0000022g0440.1	CDPK,calcium-dependent protein kinase	AGATTTATTTCGGG ATGGAAGATTTA TTTCGGGATGGA	23|0	285	AGA AGA	0|6	101	
Nitab4.5_0002658g0170.1	NB-ARC,Disease resistance protein	GTTTTTTTT GTTTTTTTT	14|0	285	GTTTTTTTTT GTTTTTTTTT	0|19	77	
Nitab4.5_0006326g0010.1	NB-ARC,Disease resistance protein	GAA GAA	19|0	285	GA GA	0|16	225	
Nitab4.5_0006326g0010.1	NB-ARC,Disease resistance protein	GAA GAA	19|0	285	GA GA	0|16	225	

## 4 Discussion

Flowering is the transition from vegetative growth to reproductive growth, which significant affect the plant growth cycle. The regulation of *FT* gene expression is influenced by CO, which further activates *SOC1*, thereby promoting flowering (Li et al., 2022a). *FLC* can inhibit flowering by weakening the expressions of *FT* and *SOC1* (Sun et al., 2020). In Arabidopsis, CO binds to the *FT* promoter and activates its expression. Conversely, the transcriptional repressor *FLC* inhibits *FT* expression, negatively regulating flowering in Arabidopsis (Tang et al., 2022). In tobacco, *NtFT1*, *NtFT2*, and *NtFT3* function as flowering repressors, whereas *NtFT4* and *NtFT5* act as flowering inducers. Overexpression of the Arabidopsis *FT* gene in tobacco markedly accelerates flowering (Dai et al., 2023). In this present study, low-temperature treatment caused both varieties flowering earlier, and M18 exhibited earlier bud formation and flowering than that of K326 under both conditions (**Figures 1A, B**). Following low-temperature treatment, the expression levels of *NtFT4* and *NtFT5* increased rapidly, with LT-M18 consistently exhibiting higher expression than that of LT-K326 (**Figures 1C, D**). There result determined that the two positive factors upregulation by low temperature maybe the direct cause of M18 earlier flowering. To further explore the potential molecular mechanisms cold induced early flowering of M18, resequencing analysis of M18 and the wild-type K326 were conducted. Compared to the reference genome, only 0.86% of SNPs and 0.26% of InDels in M18 were located within exons. Moreover, nearly half of the SNP variations in genomic exons were synonymous mutations, while over 17% of InDel variations were non-frameshift mutations. These two mutation types typically do not alter protein function. To further investigate the functions of the variant genes, GO and KEGG annotation and enrichment analyses were performed on the differential non-synonymous mutations, frameshift mutations, stop-gain and stop-loss mutations. The results showed the variant genes mainly enriched in plant reproduction, metal ion transport, plant metabolic pathways, oxidative phosphorylation and plant photosynthesis (**Figure 3B**). Furthermore, a total of 75 important candidate genes were identified, based on analyses such as gene function annotation, mutation type, and whether it is homozygous.

Notably, among these candidate genes, transcription factors including *MYB*, *bHLH*, *WD40*, *BZR1*, *WRKY*, and *bZIP*, as well as 14-3-3, MAPK, and CDPK proteins, were found to not only respond to plant flowering but also to be involved low-temperature responses in plants. *MYB* transcription factor can be induced by gibberellin, regulating flowering time and floral organ formation in plants (Li et al., 2022b). WD40 protein also play a role in regulating flowering time (Zhang et al., 2023). Heterologous expression of *PtrMYB192* in Arabidopsis negatively regulates flowering time by activating *FLC* expression (Liu et al., 2013). The bHLH protein family regulates light signaling pathways. PIF4 protein directly activates *FT* expression, promoting plant flowering. *bHLH113* form a complex inhibiting SOC1, FT, and other genes (Kumar et al., 2012). Research has found that *WRKY8*, *WRKY28*, and *WRKY71* within the *WRKY* family can directly activate the expression of flowering-related genes *FT* and *LFY* (Yu et al., 2016, Yu et al., 2018). Within the *bZIP* transcription factor family, members of the A and D subfamilies are reported to participate in plant flowering processes (Fujita et al., 2013; Yoshida et al., 2010). For example, *AtTGA9/AtbZIP21* and *AtTGA10/AtbZIP65* regulate anther development in the D-alpha family (Murmu et al., 2010), while *AtTGA8/AtPAN/AtbZIP46* controls the formation of floral organ primordia (Maier et al., 2011). Previous studies have revealed that 14-3–3 proteins play a crucial role in the regulation of flowering by florigen. In rice, the interaction between FT and FD is mediated by 14-3-3, forming the florin-activated complex (FAC) that regulates the expression of downstream flowering genes *API* and *LFY* (Taoka et al., 2011). For instance, in Arabidopsis, T-DNA insertion mutants 14-3-3µ causes late flowering (Mayfield et al., 2007). In this present study, it was found that *MYB*, *WD40*, *bHLH*, *WRKY*, *bZIP*, and 14-3–3 all exhibit SNP variations. Among these, MYB, WD40, and WRKY are present in both stop-gain and nonsynonymous SNV mutation types. bHLH and bZIP belong to the nonsynonymous SNV category, while 14-3–3 is classified as stop-gain. It is speculated that *MYB*, *bHLH*, *WRKY*, and 14-3–3 proteins in tobacco may regulate the expression of genes such as *FLC*, *SOCI*, *FT*, and *LFY*, while *bZIP* promotes the expression of genes associated with floral primordia formation (*AP1*, *LFY*, *CAL*, *FUL*, *AGL24*), collectively regulating the flowering time of tobacco. The variation of these genes maybe affect the activity or specificity of their corresponding proteins, thereby causing changes in the growth and development processes of tobacco plants.

Research has revealed that WRKY not only regulates plant flowering time but also modulates plant stress resistance through multiple pathways, including hormone signaling transduction, Ca²^+^ signaling pathways, and ion homeostasis. *MsWRKY33* activates the ethylene signaling pathway to enhance alfalfa salt tolerance (Ma et al., 2023). *AtWRKY33* undergoes synergistic phosphorylation by protein kinases CPK5/CPK6 and MPK3/MPK6, enhancing Arabidopsis resistance to biotic stress by activating Ca²^+^ signaling (Zhou et al., 2020). Class IId *WRKY* transcription factors specifically bind to the oberon (OBE) protein, directly suppressing the expression of stress-response genes such as *DREB1A, DREB1B, and DREB1C* (Du et al., 2023). For or instance, *WRKY41* inhibits *CBF/DREB1* expression in Nicotiana tabacum, thereby reducing plant frost tolerance (Wang et al., 2022). The interaction between *OsWRKY76* and *OsbHLH148* trans-activates the expression of *OsDREB1B*, enhancing rice cold tolerance (Zhang et al., 2022). Under cold stress, the binding of *VvWRKY28* to cis-acting elements promotes the expression of cold stress-related downstream genes *RAB18*, *COR15A*, *ERD10*, *PIF4*, *COR47*, and *ICS1* in Arabidopsis (Liu et al., 2022). The WRKY family in plants also regulates drought stress tolerance by mediating brassinosteroids (BR) (Han et al., 2023). Researches indicate that BES1/BZR1 not only regulates plant growth and development but also responds to biotic and abiotic stresses. For instance, BES1/BZR1 enhances the expression of genes such as *CBF* and *WRKY6* and interacts with *WRKY54* to positively regulate Arabidopsis cold tolerance (Chen et al., 2017). Genes within the WD40 family participate in the response to various abiotic stresses in multiple plant species, including Arabidopsis, rice, cucumber, peach, and walnut (Chen et al., 2022). This study found that both MAPK and CDPK belong to frameshift deletions among InDel variants. It is speculated that *WRKY* in tobacco undergoes synergistic phosphorylation by protein kinases CDPK and MAPK, thereby activating Ca²^+^ signaling. Concurrently, *BZR1* promotes the expression of *CBF* and *WRKY*, while *WRKY* interacts with *bHLH* to activate the *ICE-CBF-COR* pathway. This cascade regulates the expression of cold-tolerance genes, ultimately enhancing tobacco’s cold tolerance. These genetic variations may affect the activity or specificity of their corresponding proteins or enzymes, thereby disrupting low-temperature signal transduction processes and altering tobacco’s cold tolerance. Taken together, under low-temperature stress, mutations in *MYB, WRKY, bHLH, BZR1, WD40*,14-3-3, MAPK, and CDPK disrupt the ICE-CBF-COR pathway, altering protein activity or DNA-binding specificity. This further upregulates flowering-promoting genes *NtFT4* and *NtFT5*, accelerating the vegetative-to-reproductive transition and causing earlier flowering in M18 than in wild-type K326. In this study, our core research objective was to develop reliable molecular markers and fundamental genetic resources for routine molecular marker-assisted breeding in tobacco, thus, we focused on SNP and InDel variations. However, we acknowledge that higher-depth resequencing and experimental validation (e.g., PCR) will be adopted in our follow-up studies if we specifically explore complex SVs/CNVs and their functions.

## 5 Conclusion

This study utilized whole-genome resequencing to characterize genomic-level variation and diversity between K326 and the mutant M18, thereby investigating the potential molecular mechanisms underlying cold-induced early flowering in sensitive cultivars. Furthermore, key candidate genes from the *MYB*, *bHLH*, *WD40*, *BZR1*, *WRKY*, *bZIP*, 14-3-3, MAPK, and CDPK families were identified. In future work, conducting in-depth exploration and analysis of these mutation sites or genes and developing corresponding markers will help elucidate the molecular regulatory mechanisms underlying cold-induced early flowering in tobacco. This research will also provide important genetic resources for molecular marker-assisted breeding.

## Data Availability

The data presented in the study are deposited in Figshare: https://doi.org/10.6084/m9.figshare.32102149.
