# Supplementary material for: Whole genome resequencing analysis of tobacco K326 and its cold sensitive mutant M18
Source: Front Plant Sci. 2026 May 1;17:1829823. doi: 10.3389/fpls.2026.1829823 (PMC13176297; doi:10.3389/fpls.2026.1829823)
Supplement: Supplementary file 1 [file SupplementaryFile1.docx]

Supplementary Table S1 Quality of genome sequencing

| Sample | Raw bases(bp) | Clean bases(bp) | Effective rate(%) | Error rate(%) | Q20(%) | Q30(%) | GC content(%) |
| --- | --- | --- | --- | --- | --- | --- | --- |
| K326 | 135,695,988,300 | 134,594,705,400 | 99.13 | 0.03 | 96.55 | 91.13 | 39.48 |
| M18 | 135,687,904,200 | 134,744,239,500 | 99.31 | 0.03 | 96.51 | 90.77 | 39.09 |

Note: Q20 refers to the percentage of bases with a Phred score exceeding 20 among the total bases; Q30 denotes the percentage of bases with a Phred score exceeding 30 among the total bases.

Supplementary Table S2 Sequencing sample matching genome situation

| Sample | Mapped reads | Total reads | Mapping rate (%) | Average depth(X) | Coverage at least 1X (%) | Coverage at least 4X (%) |
| --- | --- | --- | --- | --- | --- | --- |
| K326 | 894,510,602 | 897,298,036 | 99.69 | 24.44 | 98.56 | 95.52 |
| M18 | 895,447,840 | 898,294,930 | 99.68 | 25.71 | 98.14 | 95.21 |

Note: Mapped bases: refering to the reads aligned to the reference, encompassing single-ended and double-ended alignments; Mapping rate: denoting the alignment rate, calculated as the number of reads aligned to the reference genome divided by the total reads in valid sequencing data; Average depth: determined by dividing the total number of bases aligned to the reference genome by the size of the covered genome; Coverage at least: representing genomic coverage, indicates the percentage of sites covered by at least one (1×) or four (4×) bases in the reference genome relative to the total genome length.

Supplementary Table S3 Statistics of genomic variation

|  | Category | K326 | M18 | Difference |
| --- | --- | --- | --- | --- |
| SNP | Total | 115,518,3 | 172,433,9 | 569156 |
|  | Intergenic | 112,069,5(97.01%) | 162,427,2(94.20%) | 503577 |
|  | Upstream | 548,1(0.47%) | 155,02(0.90%) | 10021 |
|  | Downstream | 522,9(0.45%) | 145,36(0.84%) | 9307 |
|  | Exonic | 573,5(1.50%) | 148,92(0.86%) | 9157 |
|  | Intronic | 173,13(0.01%) | 533,70(3.10%) | 36057 |
|  | upstream/downstream | 538(0.05%) | 996(0.06%) | 458 |
|  | Splicing | 71(0.01%) | 140(0.01%) | 69 |
|  | Others | 122(0.01%) | 633(0.04%) | 511 |
| InDel | Total | 286,674 | 360,131 | 73457 |
|  | Intergenic | 261,643(91.27%) | 321,791(89.35%) | 60148 |
|  | Upstream | 570,6(1.99%) | 881,0(2.45%) | 3104 |
|  | Downstream | 431,8(1.51%) | 670,5(1.86%) | 2387 |
|  | Exonic | 469(0.16%) | 925(0.26%) | 456 |
|  | Intronic | 141,62(4.94%) | 212,09(5.89%) | 7047 |
|  | Upstream/Downstream | 222(0.08%) | 355(0.1%) | 133 |
|  | Splicing | 29(0.01%) | 58(0.1%) | 29 |
|  | Others | 130(0.04%) | 283(0.08%) | 153 |
| SV | Total | 284,07 | 307,26 | 2319 |
|  | Intergenic | 123,56(88.48%) | 141,32(87.27%) | 1776 |
|  | Upstream | 233(1.67%) | 307(1.9%) | 74 |
|  | Downstream | 186(1.33%) | 256(1.58%) | 70 |
|  | Exonic | 435(3.11%) | 600(3.71%) | 165 |
|  | Intronic | 738(5.82%) | 880(5.43%) | 142 |
|  | Upstream/Downstream | 10(0.07%) | 9(0.06%) | -1 |
|  | Splicing | 3(0.02%) | 4(0.02%) | 1 |
|  | Others | 4(0.03%) | 5(0.03%) | 1 |
|  | INS | 73 | 75 | 2 |
|  | DEL | 129,70 | 148,82 | 1912 |
|  | INV | 922 | 1236 | 314 |
|  | ITX | 137,32 | 137,43 | 11 |
|  | CTX | 710 | 790 | 80 |
| CNV | Total | 106,307 | 106,878 | 571 |
|  | Intergenic | 312,049(97.85%) | 313,975(97.92%) | 1926 |
|  | Upstream | 967(0.3%) | 917(0.29%) | -50 |
|  | Downstream | 909(0.29%) | 910(0.28%) | 1 |
|  | Exonic | 245,0(0.77%) | 236,0(0.74%) | -90 |
|  | Intronic | 248,5(0.78%) | 239,6(0.75%) | -89 |
|  | Upstream/Downstream | 28(0.01%) | 41(0.01%) | 13 |
|  | Others | 33(0.01%) | 35(0.01%) | 2 |
|  | Gain number | 425,26 | 437,55 | 1229 |
|  | Loss number | 637,81 | 631,23 | -658 |
|  | Gain length(bp) | 125,514,805 | 127,537,253 | 2022448 |
|  | Loss length(bp) | 121,084,869 | 147,693,645 | 26608776 |

Supplementary Table S4 SNP mutation pattern and heterozygosity rate

| Sample | Total | Transition | Transvertion | Ts/Tv | Homozygosity | Heterozygosity | Het-ratio |
| --- | --- | --- | --- | --- | --- | --- | --- |
| K326 | 115,518,3 | 798,758 | 356,425 | 2.241 | 858,301 | 296,882 | 0.257 |
| M18 | 172,433,9 | 119,577,1 | 528,568 | 2.262 | 125,531,9 | 469,020 | 0.272 |


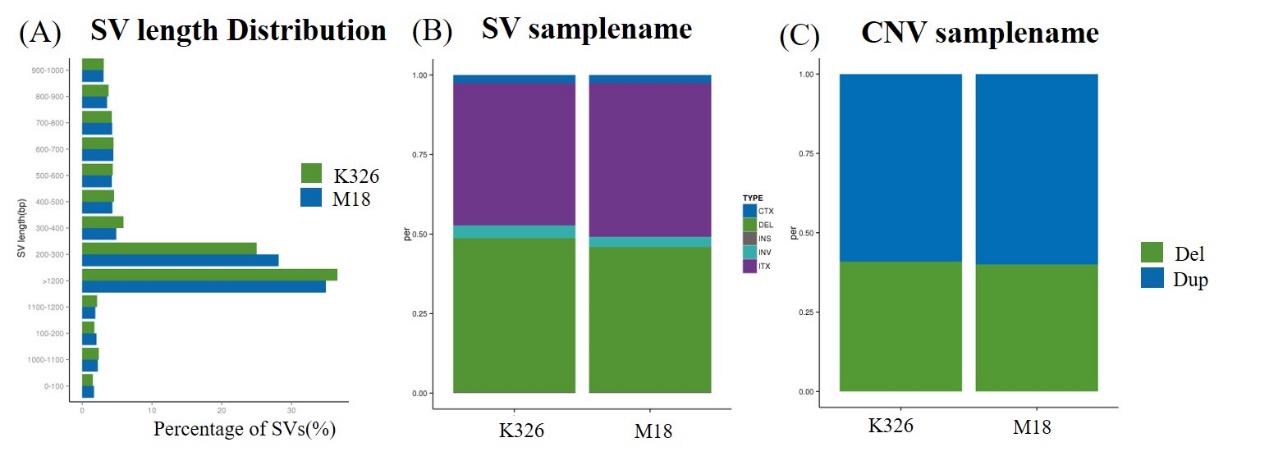


Supplementary Figure S1 SV (A,B) and CNV (C) mutation statistics.
